# CO_2_ Electroreduction Coupled with the Electrooxidation of Alcohols and Sugars to Formate: Review and Evaluation

**DOI:** 10.1002/cssc.202500478

**Published:** 2025-06-09

**Authors:** Chongyang Jiang, Zhenlei Zhang, Peter J. Deuss, Haifeng Dong, Shaojuan Zeng, Xiangping Zhang, Dulce M. Morales

**Affiliations:** ^1^ Beijing Key Laboratory of Solid State Battery and Energy Storage Process State Key Laboratory of Mesoscience and Engineering Institute of Process Engineering Chinese Academy of Sciences Beijing 100190 China; ^2^ Engineering and Technology institute Groningen (ENTEG) University of Groningen Nijenborgh 3 Groningen 9747 AG Netherlands; ^3^ State Key Laboratory of Heavy Oil Processing College of Chemical Engineering and Environment China University of Petroleum Beijing 102249 China

**Keywords:** alcohol electrooxidation, CO_2_ electroreduction, economic assessments, environmental assessments, integrated systems, sugar electrooxidation

## Abstract

Formate is a significant chemical raw material widely used in medicine, agriculture, dyes, and energy storage. The electroreduction of carbon dioxide (CO_2_) to formate has become a hot topic in the context of CO_2_ capture and utilization, which is relatively mature and industrially applicable. Currently, the oxygen evolution reaction (OER), which is the counter process at the anode of the electroreduction of CO_2_ to formate, is the cause of high energy demand. Alternatively, electrooxidation of alcohols and sugars (mono‐ and polyols) can be implemented as anode processes to decrease the energy requirements and enable the possibility of generating value‐added products on both sides of the electrolysis cell. This article reviews the literature on CO_2_ electroreduction coupled with the electrooxidation of alcohols and sugars, with formate as a target product, and analyzes factors that influence their efficiency such as catalytic materials, electrolytes, and electrolyzer designs in the coupled configuration. Additionally, the current understanding of the economic and environmental feasibility of coupling these two reactions towards formate production and upscaling deployments will be analyzed. Finally, current challenges and possible future development directions towards implementation at scale are pointed out.

## Graphical abstract

This review summarizes the latest literature on the production of formate by coupling the CO_2_ electroreduction with the electrooxidation of alcohols or sugars, covering the selection of catalysts, optimization of electrolytes, electrolyzer design and product separation. The economic and environmental impact of electroproduction of formate are analyzed, while the prospects for future developments of this coupled system are highlighted.

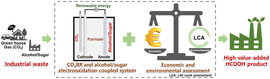

## 1 Introduction

In 2024, the concentration of carbon dioxide (CO_2_) in the atmosphere reached 420.9 ppm, which is ≈50% higher than pre‐industrial levels.^[^ ^1^ ^]^ Reducing CO_2_ emissions is crucial for mitigating environmental harm (ocean acidification, climate change, glacial melting, extreme weather events, etc.) and ensuring a sustainable future. Different technologies have been developed, including CO_2_ capture and utilization (CCU) and direct air capture (DAC).^[^ ^2^, ^3^, ^4^ ^]^ These are essential for mitigating the effects of climate change but often involve significant costs. For example, physical absorption demonstrated the lowest cost per ton of CO_2_ captured at $12.38, in comparison to chemical absorption at $48.07, membrane separation at $36.78, and pressure swing adsorption at $33.30.^[^ ^5^ ^]^ Moreover, storing the captured CO_2_ requires additional expenditures on transportation and infrastructure, which increases the financial burden of the CO_2_ sequestering process. Therefore, the conversion of CO_2_ into valuable platform molecules for the chemical production of fine chemicals and materials is key to generating revenue and thus offset the cost of CO_2_ capture.

The CO_2_ conversion pathways reported in the existing literature include the CO_2_ electroreduction, biochemical processes, and thermochemical methods.^[^ ^6^, ^7^, ^8^, ^9^, ^10^ ^]^ The CO_2_ electroreduction reaction (CO_2_RR) utilizes electrical energy to produce fuels and chemicals, such as formate, carbon monoxide, methane, ethanol, and others. Among the various possible CO_2_RR products, formate is of high relevance since its global market is expected to reach $878.7 million by 2027. Compared to traditional industrial methods for formate production such as methanol carbonylation, which require high pressure and precise catalytic conditions, the advantage of preparing formate via the CO_2_RR is that it can be carried out at room temperature and ambient pressure and can utilize electricity generated from renewable energy sources, such as solar and wind power, helping to reduce dependence on fossil fuels. In terms of industrial applications, formate can be used to synthesize medicines, pesticides, and dyes.^[^ ^11^ ^]^ Furthermore, formate has a hydrogen mass fraction of 4.4% and a volume capacity of 53 g H_2_ L^−1^, which could be directly used as a clean fuel in formate fuel cells and as a promising liquid hydrogen storage medium.^[^ ^12^, ^13^ ^]^ In summary, formate has the advantages of wide applicability, including being an excellent medium for hydrogen storage, thereby bearing high economic value.^[^ ^14^, ^15^, ^16^ ^]^ Therefore, the CO_2_ electroreduction to formate has become a relevant research focus.

The CO_2_ electroreduction reported in the existing literature is usually coupled with the oxygen evolution reaction (OER) as anode process.^[^ ^17^, ^18^, ^19^, ^20^, ^21^ ^]^ The OER shows high stability in industrial applications, producing O_2_ at high current densities without forming additional byproducts.^[^ ^22^ ^]^ However, due to the slow kinetics and the high overpotentials required for the OER, the energy costs of the overall process are considerably high.^[^ ^23^ ^]^ Additionally, there is no economic value for the oxygen produced electrochemically, which further limits the commercial application of CO_2_ electroreduction technology.^[^ ^24^, ^25^ ^]^ Production cost reduction and efficient electricity use can be addressed by considering the electrolysis process as a whole rather than as two separate half‐reactions. For this reason, researchers have begun to explore alternative anode reactions, in particular, the electrooxidation of organic compounds with oxidation potentials lower to those of the OER, with the aim of increasing the energy efficiency of the overall process.^[^ ^26^, ^27^, ^28^, ^29^ ^]^ Examples of anode reactions that are currently receiving significant attention are the electrooxidation of alcohols (such as methanol or glycerol),^[^ ^30^, ^31^ ^]^ sugars (such as glucose or xylose),^[^ ^32^, ^33^ ^]^ and of lignin and lignin‐derived compounds (such as lignosulfonates and phenol).^[^ ^34^, ^35^, ^36^ ^]^ In 2019, Na et al.^[^ ^37^ ^]^ pointed out that the electrooxidation of biomass‐derived compounds is considered to be a feasible alternative to the OER. Biomass‐derived compounds have become crucial renewable feedstocks and a sustainable substitute for fossil resources. Thus, coupling the CO_2_ electroreduction (as a cathode process) with the electrooxidation of biomass‐derived compounds (as an anode process) represents an attractive route to valorize both CO_2_ and biomass‐derived compounds, improving thereby not only the energy efficiency of the overall electrolysis system but also the economic potential due to the possibility of obtaining value‐added products from the oxidation of these organic compounds. In particular, obtaining formate as a product simultaneously on both sides of the electrochemical reactor can simplify the formate separation process, thus helping to reduce the cost of the entire production process and improve economic efficiency.

In this review, we cover innovative literature reports on CO_2_ electroreduction coupled with the electrooxidation of alcohols and sugars (mono‐ and polyols), specifically with formate as the main product of the two reactions, published in the past 5 years. The selection of cathode and anode electrode materials, the composition of the electrolyte, and other factors related to the electrolyzer are introduced in detail. By optimizing these aspects, both the reaction rate and selectivity can be enhanced, thus achieving a more efficient electrocatalytic process. In addition to this, the design of integrated systems also needs to consider requirements for industrial production. This includes not only the operating conditions of the electrolyzer but also the subsequent separation and purification processes, as these affect the long‐term performance and the energy consumption of the entire system. Finally, the economic and environmental impact of systems that couple the CO_2_RR with the electrooxidation of alcohols and sugars is discussed.

## 2 Coupling the Electrochemical Conversion of CO_2_ and Alcohols/Sugars

**Figure 1:**
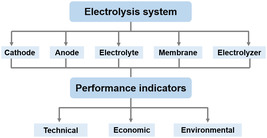
Components in the electrolysis system that influence different performance parameters for CO_2_ electroreduction coupled with alcohol/sugar electrooxidation.

Coupling the CO_2_RR with the electrooxidation of bio‐based alcohols or sugars (mono‐ and polyols) to formate requires the careful design of electrodes (anode and cathode), electrolytes, membranes, and electrolyzers to achieve excellent electrochemical performance and economic/environmental advantages. These are usually assessed by means of performance indicators of three types: technical, economic, and environmental (**Figure** **1**). The first type, concerning the technical evaluation of the coupled electrochemical system, as well as the factors affecting the two electrochemical conversions towards formate production, will be introduced in this section.

### 2.1 CO_2_ Electroreduction to Formate

The CO_2_RR process to formate is energetically challenging because CO_2_ is a highly stable molecule, and there are various CO_2_ electroreduction products with similar electroreduction potentials.^[^ ^38^ ^]^ The reaction involves four main steps: 1) gaseous CO_2_ dissolves in the electrolyte, 2) CO_2_ diffuses to and adsorbs onto the electrode surface, 3) CO_2_ obtains electrons and protons to form formate, and 4) formate desorbs from the active sites and diffuses into the electrolyte.^[^ ^39^ ^]^ According to the literature, the initial step is the transfer of an electron to the CO_2_ molecule to form *CO_2_^−^ (* indicates the adsorption site). Next, the adsorbed *CO_2_^−^ can combine with protons from the electrolyte to form *OCHO or *COOH. If the C atom in *CO_2_^−^ is adsorbed on the catalytic active site, an O atom is protonated to form *COOH, which is eventually released to form CO or further reduced to form different hydrocarbons or alcohols. If instead the O atom in *CO_2_^−^ is adsorbed on the catalytic active site, the C atom is protonated to form *OCHO and formate is obtained.^[^ ^40^ ^]^

**Figure 2:**
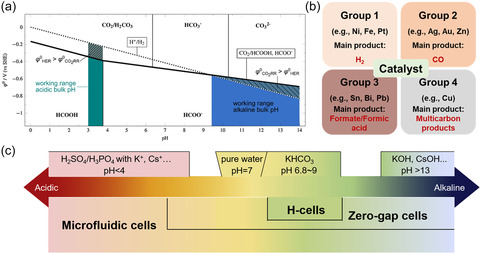
a) Pourbaix diagram for CO_2_ electroreduction to formic acid/formate; the working pH ranges of acidic and alkaline electrolytes are indicated in teal and blue, respectively; Reprinted from Ref. [42] Copyright 2022, with permission from Elsevier. b) Four groups of metal catalysts for the HER and for the CO_2_ electroreduction to formate, CO and hydrocarbons/oxygenates. c) Type of electrolytes typically reported for CO_2_ electroreduction; Reprinted from Ref. [59] Copyright 2023, with permission from Elsevier.

It is worth noting that both HCOOH (formic acid) and HCOO^−^ (formate) can be produced by CO_2_ electroreduction. Formic acid is formed when the electrolyte has a pH below 3.75, although this pathway is not thermodynamically favored. When the pH of the electrolyte is above 3.75, formate is obtained. It is also important to consider that, when the pH of the electrolyte is higher than 7, the potential for the electroreduction of CO_2_ to formate is lower than that of the competing hydrogen evolution reaction (HER). Thus, increasing the alkalinity of the electrolyte can promote the CO_2_ electroreduction to formate (**Figure** **2**a).^[^ ^41^, ^42^ ^]^ Additionally, electrolysis in alkaline media makes it possible to employ earth‐abundant, low‐cost materials as electrocatalysts, in contrast to electrolysis in acidic media which require using noble metals due to their high corrosion resistance. For these two reasons, in this review, we focus on alkaline electrolysis and, particularly, the production of formate rather than formic acid. The parameters commonly used to assess the technical performance of an electrolysis system (Figure 1) are related to the activity (overpotential, current density, and conversion), selectivity (Faradaic efficiency (FE) and/or yield towards certain product), and stability (performance over time).

The selectivity of the CO_2_RR depends on the relative bonding strengths of the reaction intermediates (*COOH, *OCHO, *CO, and *H) on the catalyst surface, and it is typically determined by product analysis techniques including high‐performance liquid chromatography (HPLC), gas chromatography (GC), nuclear magnetic resonance (NMR). Therefore, the nature of the catalysts determines the selectivity, and can be divided into four groups according to the different adsorption capacities of intermediates on their surfaces (Figure 2b). The first group of catalysts includes Ni, Fe, and Pt, which exhibit low overpotentials towards the HER and moderate adsorption ability for *H, thereby favoring the HER. The second group, which includes Ag, Pd, Au, and Zn, preferentially adsorbs *COOH from a thermodynamic perspective, thus generally favoring the formation of CO as product. The third group of catalysts includes Hg, Cd, Pb, Sn, Bi, and In, which thermodynamically facilitate the adsorption of *OCHO, resulting in the formation of mainly formate. The fourth group is Cu, which can stabilize the *CO and *COOH intermediates, and thus can favor the formation of multicarbon products.^[^ ^43^ ^]^

In the case of the conversion of CO_2_ to formate, the nature of the electrocatalyst also affects the activation and conversion efficiency of the CO_2_ molecules. In particular, Bi and Sn‐based catalysts have attracted widespread attention due to their low cost. Bi and Sn‐based catalysts also have a relatively high oxygen affinity and a weak hydrogen affinity. The latter is beneficial as it can help suppress the competing HER, while the former favors the formation of the oxygen‐bond intermediates, such as *OCHO, thus having a preferable selectivity towards formate production. For example, Chai et al.^[^ ^44^ ^]^ synthesized porous Bi, which showed a FE over 91% towards formate in a broad potential range from −0.75 to −1.1 V vs. the reversible hydrogen electrode (RHE) in an H‐type electrochemical cell. Besides, Qu et al.^[^ ^45^ ^]^ also reported that carbon nanotubes‐supported Sn_3_O_4_ electrocatalyst exhibited a maximum FE of 91% towards formate at −0.79 V vs. RHE. In addition to this, a large number of literature reports have shown modification of Bi‐ and Sn‐based catalysts through various strategies to improve their catalytic activity and stability. For example, the electronic structure and properties of their catalytic sites can be optimized by alloying, doping, constructing heterostructures, or designing specific nanostructures.^[^ ^16^ ^]^ Some review articles systematically summarize these modification strategies and explore the opportunities and challenges of cathode‐side catalysts in CO_2_RR.^[^ ^46^, ^47^, ^48^, ^49^, ^50^, ^51^, ^52^, ^53^ ^]^

As an ion transport medium in the electrolysis process, the electrolyte also has an important impact on the efficiency of the CO_2_RR process.^[^ ^54^, ^55^, ^56^ ^]^ The selection of a suitable electrolyte often depends on the reactor design (Figure 2c). For instance, in an H‐type electrochemical cell, the electrolyte employed for the CO_2_ electroreduction side (catholyte) is typically aqueous KHCO_3_, which serves as the proton and carbon source at the same time.^[^ ^57^, ^58^ ^]^ When using a flow electrolyzer, 1 M KOH is often used to ensure high conductivity, thereby overcoming resistance limitations in the electrochemical cell and reducing the overall cell voltage.^[^ ^59^, ^60^, ^61^, ^62^ ^]^ On the other hand, the formation of H_2_ is more favored under lower pH values, while higher pH values could suppress HER and boost the CO_2_RR selectivity towards formate. Gorthy et al.^[^ ^63^ ^]^ used density functional theory (DFT) to calculate the influence of the KOH concentration on the CO_2_ electroreduction. It was found that increasing the KOH concentration reduced the energy barrier for the electroreduction CO_2_ and thus improved the current density. For the influence of electrolyte on CO_2_ electroreduction performance, please refer to the following literature.^[^ ^64^, ^65^, ^66^ ^]^

Another important component is the ion exchange membrane, which is used to separate the cathode chamber and the anode chamber while facilitating selective ionic transfer and preventing reagent and product crossover. The types of membranes include anion exchange membrane (AEM), cation exchange membrane (CEM), and bipolar exchange membrane (BPM). Most reported lab‐scale electrochemical reactors use Nafion membranes, among which Nafion 117 is the most widely used CEM. AEM and CEM are cost‐effective, stable, and easy to manufacture; however, both face challenges such as high product crossover and high pH gradient across the membrane.^[^ ^67^, ^68^, ^69^ ^]^ These disadvantages can be addressed by using a BPM, which consist of a layered ion‐exchange structure composed of a cation‐selective layer and an anion‐selective layer. BPMs can effectively preserve a stable pH level across its structure by selectively facilitating the movement of anions and cations.^[^ ^70^ ^]^ However, BPMs are more expensive, have a shorter lifetime, and suffer from lower stability. The short lifespans of BPMs are largely attributed to the crossover of bicarbonate ions, which converts to CO_2_ leading to membrane swelling.^[^ ^71^, ^72^ ^]^ For a detailed comparison of different membrane types, see **Table** **1**.^[^ ^73^, ^74^ ^]^

**Table 1:** Comparative analysis of different types of membranes.

Membrane type	Ion selectivity	Operating pH	Conductivity	Thickness	Advantages	Challenges
AEM	OH	Alkaline	20–80 mS cm^−1^ at room temperature	25–100 μm	Reduced catalyst degradation in alkaline environment	Vulnerable to carbonate buildup; long‐term stability issues in high‐pH environments
CEM	H+	Acidic	50–100 mS cm^−1^ at 80 °C	20–50 μm	High ionic conductivity; effective in suppressing side reactions; suitable for compact designs	Limited stability in acidic conditions; catalyst options limited by acidic environment; CO_2_ crossover
BPM	H^+^ and OH^−^	Acidic/ alkaline	5–20 mS cm^−1^ per layer (H^+^ and OH^−^ layers)	50–200 μm (combined thickness)	Allows local pH tuning (acidic at one electrode, alkaline at the other); improved selectivity	Water dissociation increases energy loss; sensitivity to ion imbalance and membrane swelling

The design of the electrolyzer is one of the key factors in improving the efficiency of the CO_2_RR. H‐type electrolyzers, which are typically used for lab‐scale investigations, have been gradually replaced by flow electrolyzers due to the enhanced mass transfer efficiency of the latter. For example, using gas diffusion electrodes (GDEs) and membrane electrode assemblies (MEAs) can significantly improve the mass transfer efficiency of CO_2_ and the current density of the electrolyzer by providing a larger gas–liquid contact area.^[^ ^75^, ^76^, ^77^, ^78^, ^79^ ^]^ While this review does not delve into research advancements related to electrocatalysts, electrolytes, membranes, and electrolyzers for the CO_2_ electroreduction process, we refer the readers to numerous studies that document these aspects in detail.^[^ ^64^, ^65^, ^75^, ^76^, ^77^, ^78^, ^79^, ^80^, ^81^, ^82^ ^]^

### 2.2 Electrooxidation of Alcohols and Sugars to Formate

The electrooxidation of alcohols and sugars (mono‐ and polyols) is considered as an environmentally friendly and sustainable method for the production of formate.^[^ ^83^ ^]^ For this anode reaction, the performance indicators are similar to those of the CO_2_RR, considering the need for high activity (high current density at low overpotentials), high selectivity (high Faradaic efficiencies towards the target product), and sufficient long‐term stability. In this section, the effects of electrocatalysts, electrolytes, and electrolyzer design on the electrolysis process are discussed in detail. Furthermore, we discuss formate separation as an important aspect to consider for the economic viability of the overall process.

#### 2.2.1 Electrooxidation of Alcohols and Sugars as an Alternative Anode Reaction

As discussed in Section 1, the OER, which is typically the anode reaction that counters the CO_2_RR, is a sluggish process that requires a large energy input. Therefore, implementing alternative anode reactions is a promising route to enhance the energy efficiency of the overall electrolysis process.^[^ ^29^ ^]^ When selecting an anode reaction to replace the OER, it is crucial to ensure that its electrooxidation potential is lower than that of the OER.^[^ ^84^ ^]^ Additionally, the economic feasibility of the process could be further enhanced if the electrooxidation process selectively leads to the formation of high‐value chemicals.^[^ ^85^, ^86^, ^87^, ^88^, ^89^ ^]^ **Table** **2** lists the electrochemical potentials of the electrooxidation of various alcohols and sugars.^[^ ^90^ ^]^ It can be seen that the electrochemical potential of the OER is substantially higher than those of the listed organic compounds, indicating that the electrooxidation of water is less thermodynamically favorable than those of the organic compounds. Therefore, a reasonable design of a coupled reaction system with low energy consumption to obtain high value‐added products could make the two‐reaction system more economically feasible.

**Table 2:** Electrochemical potentials of typical anodic oxidation reactions involving alcohols and sugars.^[^ 37, 118 ^]^

Reactants	Target product	Anodic reaction	*E* ^0^ (V vs. RHE)
H_2_O	O_2_	2H_2_O → O_2_ + 4H^+^ + 4e^−^	1.23
Methanol	Formic acid	CH_4_O + H_2_O → HCOOH + 4H^+^ + 4e^−^	−0.258
Methanol	Formaldehyde	CH_4_O → CH_2_O + 2 H^+^ + 2e^−^	0.465
Xylose	Formic acid	C_5_H_10_O_5_ + 5H_2_O → 5HCOOH + 10 H^+^ + 10e^−^	0.619
Glucose	Gluconic acid	C_6_H_12_O_6_ + 2OH^−^ → C_6_H_12_O_7_ + H_2_O + 2e^−^	0.948
Ethanol	Acetic acid	C_2_H_6_O + H_2_O → C_2_H_4_O_2_ + 4H^+^ + 4e^−^	−0.334
Ethanol	Acetaldehyde	C_2_H_6_O → C_2_H_4_O + 2 H^+^ + 2e^−^	0.193
Ethanol	Ethyl acetate	2C_2_H_6_O → C_4_H_8_O_2_ + 4H^+^ + 4e^−^	−0.208
Glycerol	Formic acid	C_3_H_8_O_3_ + 8OH^−^→ 3HCOOH + 5H_2_O + 8e^−^	−0.703
Glycerol	Lactic acid	C_3_H_8_O_3_ → C_3_H_6_O_3_ + 2 H^+^ + 2e^−^	0.041
Furfural alcohol	2‐Furoic acid	C_5_H_6_O_2_ + H_2_O → C_5_H_4_O_3_ + 4H^+^ + 2e^−^	−0.515
5‐Hydromethylfurfural	2,5‐Furandicarboxylicacid	C_6_H_6_O_3_ + 2H_2_ O→ C_6_H_4_O_5_ + 6 H^+^ + 6e^−^	−0.78

The electrooxidation of hydroxyl groups in alcohols and sugars in alkaline media results in the formation of ketones/aldehydes, carboxylates, and/or carbonate. Compared with other valuable products such as lactate and acetate, formate has broad application prospects in fuel cells and energy storage systems. In addition to this, literature shows that formate can be obtained with a very high selectivity compared to other products. Obtaining formate as the sole product of both half‐reactions of the electrolysis cell can reduce the complexity and operating costs of subsequent processing (vide infra). **Table** **3** lists the relevant performance data reported in existing literature concerning the CO_2_RR coupled with electrooxidation of alcohols or sugars to produce formate.

**Table 3:** Summary table of electroreduction of CO_2_ coupled with the electrooxidation of alcohols and sugars to formate.

Cathode catalyst	Catholyte	FE towards formate at the cathode [%]	Anode catalyst	Anolyte	FE towards formate at the anode [%]	Voltage [V]	Current density [mA cm^−2^]	Type of electrolyzer	Membrane	Duration of stability tests	Reference
Bi_2_O_2_CO_3_‐CNTa)	1.0 M KOH	97.9	NiCo_2_O_4_‐CFPb)	1.0 M KOH and 0.2 M Glucose	98.4	2.4	≈80	Flow cell	AEMv)	32 h	[95]
Bi/GaN/Si	0.5 M KHCO_3_	91	NiOOH/α‐Fe_2_O_3_	1.0 M KOH and 0.2 M Glucose	85.2	1.2	≈1.7	H‐type	BPMw)	80 h	[103]
Bi‐enec)	0.5 M KHCO_3_	>90	Ni‐NF‐Afd)	1.0 M KOH and 0.5 M CH_3_OH	>90	2.13	10	H‐type	BPM	40 min	[104]
Li‐Bi_2_S_3_	1.0 M KOH	>80	Au/NiOOH@Ni	1.0 M KOH and 1.0 M CH_3_OH	>80	1.0	200	Flow cell	AEM	20 h	[31]
BiO_2_	0.5 M KHCO_3_	94.22	CuS	1.0 M KOH and 1.0 M CH_3_OH	93.46	2.8	10	H‐type	PEMx)	4.4 h	[105]
3D Bi‐ene‐A/CMe)	0.5 M KHCO_3_	>90	Ni(OH)_2_/NFf)	1.0 M KOH and 0.5 M CH_3_OH	>90	2.303	10	H‐type	BPM	1 h	[190]
Bi	1.0 M KOH	≈100	Ni(OH)_2_	1.0 M KOH and 0.5 M CH_3_OH	≈100	3.0	100	Flow cell	AEM	20 h	[106]
BiPO_4_	1.0 M KOH	>90	S–NiCo‐LDHg)	1.0 M KOH and 1.0 M CH_3_OH	>90	2.06	100	Flow cell	AEM	40 min	[191]
mSnO_2_/CCh)	1 M KHCO_3_	80.5	CuONS/CFi)	1.0 M KOH and 1.0 M CH_3_OH	91.3	0.93	10	Flow cell	–	–	[192]
Pd/MnFe_2_O_4_ NSsj)	2.0 M KHCO_3_	96.9	Pd/MnFe_2_O_4_ NSs	0.5 M KOH and 0.4 M CH_3_OH	97.5	1.0	227	H‐type	PEM	30 h	[193]
Bi‐NSs	0.5 M KHCO_3_	>90	Co(OH)_2_@HOS/CPk)	1.0 M KOH and 6.0 M CH_3_OH	>95	2.44	10	H‐type	BPM	12 h	[194]
HOD‐Cul)	0.5 M KHCO_3_	–	HOD‐CuOm)	1.0 M KOH and 1.0 M CH_3_OH	78	2.18	10	H‐type	BPM	30 h	[195]
InS NRsn)	1.0 M KOH	94.2	(oxy)hydroxide@np‐Ni_3_Po)	1.0 M KOH and 0.5 M CH_3_OH	98.1	2.286	50	H‐type	BPM	32 h	[196]
CuSn alloy	0.5 M KHCO_3_	93.2	CuSn alloy	1.0 M KOH and 1.0 M CH_3_OH	99.1	3.23	100	H‐type	PEM	300 min	[23]
CuCo_2_Se_4_	0.3 M NaHCO_3_	92.5	CuCo_2_Se_4_	1.0 M KOH and 1.0 M C_2_H_5_OH	84.35	0.67	15	H‐type	PEM	100 h	[197]
V_o_‐BOC‐NSp)	1.0 M KOH	>90	Ni_3_N‐Co_3_N/NF	1.0 M KOH and 0.5 M CH_3_OH	>90	2.27	50	Flow cell	AEM	–	[198]
BiOI	0.5 M KHCO_3_	92	Ni_0.33_Co_0.67_(OH)_2_@HOS/NFq)	1.0 M KOH and 0.1 M glycerol	90	1.9	22.4	Flow cell	PEM	36 h	[199]
BiOBr	1.0 M KOH	≈92	Ni_x_B	1.0 M KOH and 1.0 M glycerol	58	1.2	25	Flow cell	PEM	2.5 h	[168]
Bi/C	0.5 M KCl and 0.45 M KHCO_3_	>60	Ni‐Co	1.0 M KOH and 1.0 M glycerol	95.1	3.4	45	Flow cell	PEM	–	[200]
Ag/BOCr)	0.5 M KHCO_3_	>50	CoP	1.0 M KOH and 0.05 M glycerol	>50	2.0	50	Flow cell	AEM	–	[201]
In Bi NP	0.5 M KHCO_3_	>80	Ni_3_S_2_/NF	1.0 M KOH and 0.05 M glycerol	>80	≈5	50	Flow cell	BPM	24 h	[70]
Tin nano powder	0.5 M KHCO_3_	74	Porous platinum	1.0 M KOH and 2.0 M glycerol	30	≈4.5	50	Flow cell	CEM^y)^	5 h	[202]
Bi/C	0.5 M KCl and 0.45 M KHCO_3_	85	Pt/C	1.0 M KOH and 1.0 M glycerol	79	3.46	45	Flow cell	PEM		[203]
Bi Nanoparticles	Humidified CO_2_	72.9	Ni NP on C microfibers	1.0 M KOH and 1.0 M glycerol	38.6	3.59	45	Flow cell	PEM	5.5 h	[204]
In_2_O_3_	1.0 M KOH	96.2	NiV	1.0 M KOH and 0.1 M glycerol	92.3	5.87	300	Flow cell	CEM	48 h	[205]
Bi_2_S_3_	Humidified CO_2_	≈90	NiCo_2_O_4_/NF	0.5 M KOH and 0.2 M glycerol	≈90	1.93	50	Flow cell	AEM	10 cycles	[206]
BOC@rGO^s)^	0.1 M KHCO_3_	≈80	CuCoO@rGo	1.0 M KOH and 0.1 M ethylene glycol	≈70	1.9	10	H‐type	AEM	3600 s	[207]
Bi/Bi_2_O_3_	1.0 M KOH	86	Ni(OH)_2_‐Vo	1.0 M KOH and ethylene glycol	91	2.7	100	Flow cell	PEM	15000 s	[208]
SnO_2_/CC^t)^	1 M NaHCO_3_	≈70	NiCo_2_O_4_	1.0 M NaOH and 0.1 M ethylene glycol	≈80	1.9	20	H‐type	AEM	2 h	[209]
Cu(F)@Pb‐SnOu)	0.5 M KHCO_3_	Production rate: 19.1 μmol h^−1^	Cu(F)@CuO@Ni(OH)_2_	1.0 M KOH and 0.1 M ethylene glycol	Production rate: 43.2 μmol h^−1^	1.63	10	H‐type	PEM	24 h	[210]

a) CNT: carbon nanotube;

b) CFP: carbon fiber paper;

c) Bi‐ene: bismuthenes;

d) NF‐Af: nickle foam nanosheet arrays;

e) Bi‐ene‐A/CM: 3D open network of interconnected bismuthene arrays on copper mesh;

f) NF: Ni foam;

g) LDH: layered double hydroxide;

h) mSnO_2_/CC: mesoporous SnO_2_ grown on carbon cloth;

i) CuONS/CF: CuO nanosheets grown on copper foam;

j) NSs: nanosheets;

k) HOS/CP: hydroxysulfide nanosheets on carbon paper;

l) HOD‐Cu: Cu(OH)_2_‐derived Cu;

m) HOD‐CuO: Cu(OH)_2_‐derived CuO;

n) NRs: nanrods;

o) np: nanoporous;

p) Vo‐BOC‐NS: ultrathin Bi_2_O_2_CO_3_ nanosheets with abundant oxygen vacancy;

q) Ni_0.33_Co_0.67_(OH)_2_@HOS/NF: nickel–cobalt hydroxide nanoneedle catalyst supported on Ni foam;

r) BOC: bismuth oxide carbonate;

s) rGO: reduced graphene oxide;

t) CC: carbon cloth;

u) Cu(F): Cu foam;

v) AEM: anion exchange membrane;

w) BPM: bipolar exchange membrane;

x) PEM: proton exchange membrane;

y) CEM: cation exchange membrane.

Another important criterion for selecting electrooxidation compounds is the price and availability of the substrate. An example of this are alcohols, such as methanol (which can be produced from syngas via the gasification of lignocellulosic biomass or by the conversion of biogas) and glycerol (which is a byproduct of the production of biodiesel from vegetable oils and animal fats).^[^ ^91^, ^92^ ^]^ Both alcohols, methanol and glycerol, can be electrooxidized to form formate, thereby enhancing the high‐value utilization of biomass resources. Additionally, the raw material prices for glycerol (approximately $170 ton^−1^) and methanol (approximately $300 ton^−1^) are relatively low, whereas the market price of formate is around $600 ton^−1^ (note that these are wholesale prices for bulk commodities, and additional costs such as purity adjustments and logistics should be considered).^[^ ^93^ ^]^ This indicates that using methanol and glycerol as feedstocks for the electrooxidation process can substantially increase the economic value of the overall process. At the same time, it is possible to obtain formate from methanol and glycerol electrooxidation with a high selectivity and current density.^[^ ^94^ ^]^ For example, Liu et al.^[^ ^31^ ^]^ reported the performance of Au/NiOOH@Ni heterojunction electrode as a electrocatalyst for the methanol electrooxidation reaction efficiently coupled with CO_2_ electroreduction to formate at an ampere‐level current density. The anode catalyst (Au/NiOOH@Ni) showed ≈100% FE towards formate in the current density range from 200 to 1200 mA cm^−2^. In situ Raman investigations and theoretical calculations revealed that Au/NiOOH heterojunctions promote high‐active Ni^3+/4+^OOH, which continuously provides abundant active sites for methanol electrooxidation.

Sugars, such as glucose or fructose, are also examples of viable compounds for the electrooxidation reaction. The electrooxidation of sugars coupled to the CO_2_RR is scarcely reported in the literature. An example is the work of Zhao et al.^[^ ^95^ ^]^ who demonstrated the CO_2_ electroreduction coupled with the glucose electrooxidation reaction (CO_2_RR//GEOR) to simultaneously produce formate in the two half‐cells. The CO_2_RR//GEOR system exhibited high FE towards formate (FE_CO2RR_  > 90%; FE_GEOR_ > 90%) in the wide cell voltage range from 1.8 to 2.4 V, achieving ≈33.3% energy saving compared to CO_2_RR//OER with a high formate yield of 0.92 mol g^−1^ h^−1^ at 100 mA cm^−2^. Meanwhile, the literature on electrooxidation of sugars as a reaction alternative to the OER is vast when coupled with the HER (instead of the CO_2_ electroreduction) as the cathode process.^[^ ^96^ ^]^ For instance, Liu et al.^[^ ^97^ ^]^ reported that, for xylose electrooxidation coupled with the HER, the potential required to achieve a current density of 100 mA cm^−2^ is 290 mV lower than what is required by the OER.

Other alcohols such as 5‐hydromethylfurfural (5‐HMF) and furfural alcohol also have important application potential in the field of electrooxidation conversion, which can be converted into high value‐added chemicals such as 2,5‐furandicarboxylic acid (FDCA) and 2‐furoic acid. FDCA and 2‐furoic acid have a wide range of applications in bioplastics, medicines, fragrances, and pesticides.^[^ ^98^, ^99^, ^100^, ^101^, ^102^ ^]^ In general, the combination of the CO_2_RR and the electrooxidation of alcohols and sugars is an emerging field that requires further investigation and optimization.

#### 2.2.2 Electrocatalysts and Electrolytes for the Electrooxidation of Alcohols and Sugars

**Figure 3:**
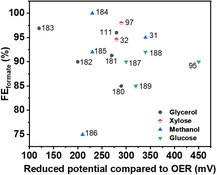
FE towards formate (FE_formate_) and decrease in electrode potential compared to the OER obtained from electrolysis of glycerol, xylose, methanol, and glucose.^[^ 31, 32, 95, 97, 111, 180, 181, 182, 183, 184, 185, 186, 187, 188, 189 ^]^

Transition metal‐based catalysts have attracted widespread attention due to their high earth‐abundance, environmental friendliness, and low cost compared to noble metal‐based materials. Particularly, Ni‐ and Co‐based catalysts have been investigated as electrocatalysts for the electrooxidation of mono‐ and polyols in alkaline media, reporting selectivities close to 100% towards formate (**Figure** **3**),^[^ ^95^, ^103^, ^104^, ^105^, ^106^ ^]^ while they can be easily modified by doping, alloying and surface engineering to further improve their activity, selectivity and stability.^[^ ^107^, ^108^, ^109^ ^]^

Ni‐based materials are considered among the most promising catalysts for these reactions due to their good electrooxidation properties, which also have significant activity and stability in alkaline electrolyte. Taking the electrooxidation of methanol as an example, Luo et al.^[^ ^110^ ^]^ prepared a hierarchical nickel oxide array on nickel foam (NiO NS@NW NF) and evaluated its performance in an alkaline electrolyte consisting of 1 M KOH and 0.5 M methanol. The results demonstrated that at a potential of 1.62 V vs. the RHE, a current density of 89 mA cm^−2^ was achieved, while in the methanol‐free electrolyte, the current density was ≈15 mA cm^−2^. Additionally, the NiO NS@NW NF‐based electrode maintained a high stability over 1000 voltametric cycles. In the case of the glycerol electrooxidation reaction, Ni‐based catalysts exhibit excellent electrooxidation performance in alkaline electrolyte (e.g., 1 M KOH) compared to other earth‐abundant transition metals, such as Fe, Mn, or Co. Morales et al.^[^ ^111^ ^]^ found that multiwalled carbon nanotube (MWCNT)‐supported Ni oxide nanoparticles only need 1.31 V vs. RHE to achieve a current density of 10 mA cm^−2^ in the presence of 1 M glycerol, while analogous Fe, Mn, and Co‐based catalysts require potentials of 1.55, 1.66, and 1.49 V vs. RHE, respectively, to achieve the same current density. In addition to this, the applied potential required for the OER to achieve 10 mA cm^−2^ was 1.59 V vs. RHE. The high electrooxidation performance exhibited by Ni‐based metals is attributed to the conversion of Ni to Ni^3+/4+^OOH during electrooxidation, which continuously provides abundant active sites.^[^ ^31^, ^112^ ^]^ These results show that Ni‐based catalysts can effectively catalyze alcohol/sugar electrooxidation at lower potentials and show higher electrooxidation activity compared to other metals, giving a significant advantage as an alternative to the OER in electrocatalytic applications.

Compared to monometallic Ni‐based catalysts, materials that incorporate Ni and a second transition metal, particularly Co, often exhibit enhanced performance towards the electrooxidation of alcohols and sugars.^[^ ^113^, ^114^, ^115^ ^]^ For instance, Ko et al.^[^ ^114^ ^]^ designed core‐shell‐like NiCo_2_O_4_ supported onto MWCNTs. The bimetallic oxide nanoparticles were homogeneously distributed on the surface of the MWCNTs, which exhibited an excellent current density of 327 mA cm^−2^ at an applied potential of 0.657 V vs. Ag|AgCl|KCl (saturated), measured in 1.0 M KOH solution containing 5.0 M methanol, while the current density corresponding to the OER (in the absence of methanol) was about ≈30 mA cm^−2^ at the same potential. Another example is the report by Braun et al.^[^ ^116^ ^]^ on polybenzoxazine‐embedded cobalt nickel boride (CoNiB), as an electrocatalyst for the glycerol electrooxidation in alkaline solutions. It was found that in 1 M KOH solution, the OER current of CoNiB increased exponentially at potentials >1.5 V vs. RHE, while in glycerol‐containing electrolytes, the increase in current density was observed at much lower potentials (around 1.32 V vs. RHE) at a current density of 10 mA cm^−2^. The difference in electrode potentials measured for the OER and for the glycerol oxidation reaction (GOR) measured at 10 mA cm^−2^ (ΔE) can be used as a measure of the energy saved by replacing the OER with the GOR. CoNiB, for instance, had a ΔE value of 224 ± 6 mV. In addition to this, Li et al.^[^ ^117^ ^]^ reported nickel‐molybdenum‐nitride nanoplates loaded on carbon fiber cloth as a GOR electrocatalyst to produce formate. In addition to this, the electrooxidation of glycerol into high‐value‐added formate with 95% selectivity was demonstrated. Owing to the much favorable thermodynamics of the glycerol electrooxidation and favorable kinetics compared to the OER, the two‐electrode configuration only required a low cell voltage of 1.36 V at a current density of 10 mA cm^−2^, which was 260 mV lower than that required by conventional water electrolysis with the OER as anode reaction.

Producing formate with high selectivity and high yield are important targets in the electrooxidation of alcohols and sugars.^[^ ^89^ ^]^ This implies that once formate is produced, its further oxidation should be prevented during the electrolysis process.^[^ ^70^, ^95^ ^]^ Zhao et al.^[^ ^95^ ^]^ analyzed ^13^C NMR spectra of electrolyte samples collected at different reaction times and found that formate was the only product from the electrooxidation of glucose during the first 2 h of electrolysis. However, when glucose was almost consumed (≈10 h), carbonate products appeared, indicating that formate was further oxidized. As the reaction time increased (≈25 h), carbonate was the only product. This was also observed during the electrooxidation of glycerol on a Ni oxide‐based catalyst, particularly when the electrode potential was increased.^[^ ^111^ ^]^ It is thus important that the anode catalyst is carefully designed and that the electrolysis conditions are optimized to prevent the full oxidation of alcohols and sugars to carbonate. Relevant catalyst design strategies can be referred to the following literature.^[^ ^26^, ^90^, ^118^ ^]^

**Figure 4:**
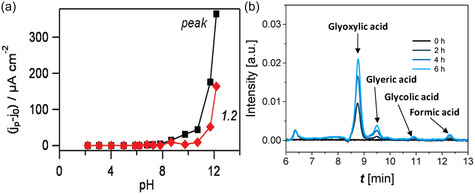
a) Current density *j*_P_ for the electrooxidation of 0.5 M ethanol on a gold electrode at peak potential (black curve) or at 1.2 V vs. RHE (red curve) plotted against the pH of the electrolyte using 0.1 M phosphate buffer solution; Reprinted with permission from Ref. [127] Copyright 2011, American Chemical Society. b) HPLC chromatograms recorded as a function of time corresponding to 6 h aging of 0.1 M glucose in 0.1 M NaOH. Reprinted with permission from Ref. [130] under a CC BY 4.0 license.

In addition to the electrode (comprising both the electrocatalytic material and the catalyst support), the electrolyte composition also plays an important role in the performance of the electrolysis system. 1 M KOH is usually used as anolyte in combination with the corresponding alcohol or sugar that will be oxidized,^[^ ^119^, ^120^, ^121^, ^122^, ^123^, ^124^, ^125^ ^]^ and using different electrolytes often leads to substantially different performances. For instance, Ruiz‐Camacho et al.^[^ ^126^ ^]^ evaluated the performance of Pt‐Ag/C catalyst towards the methanol electrooxidation in two electrolytes (0.5 M H_2_SO_4_ and 0.5 M KOH) in the presence of 0.5 M methanol. The results showed that the maximum current density in 0.5 M KOH solution was 164.6 mA cm^−2^ at 0.03 V vs. RHE, which was higher than the maximum current density measured in 0.5 M H_2_SO_4_ solution (17.5 mA cm^−2^ at 0.86 V vs. RHE). Additionally, chronoamperometric measurements also indicated that during the electrooxidation of methanol in the alkaline electrolyte, higher current densities were achieved compared to the acid electrolyte. Kwon et al.^[^ ^127^ ^]^ proposed that the initial deprotonation step (“alpha” proton transfer) in alcohol electrooxidation is base‐catalyzed, with the catalyst playing no essential role. The activity towards the electrooxidation of an alcohol varies in a highly nonlinear fashion with pH, reaching an optimal value when the solution pH is approximately close to the pKa of the alcohol (**Figure** **4**a).^[^ ^128^ ^]^ Also, sugars can self‐dissociate in alkaline media (e.g., in 0.1 M NaOH).^[^ ^129^ ^]^ For instance, Rafaïdeen et al.^[^ ^130^ ^]^ found that 0.1 M glucose aged for 6 h in 0.1 M NaOH without the presence of a catalyst, thereby producing glyoxylate, glycerate, and glycolate (Figure 4b), indicating that the hydroxide present in the solution can promote the initial deprotonation through acid‐base equilibrium.

### 2.3 Electrochemical Reactor Configuration

**Figure 5:**
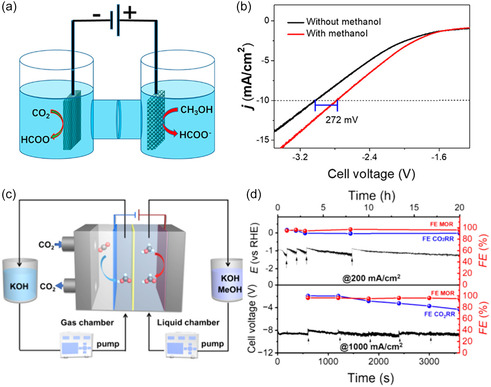
a) Illustration of CO_2_ electroreduction coupled with the methanol electrooxidation reaction to produce formate in an H‐type electrolysis cell. b) Linear sweep voltammograms of the electrolysis cell using BiO_2_ as anode and CuS as cathode, with and without the addition of 1.0 M methanol, in 1 M KOH; Reprinted with permission from Ref. [105] Copyright 2022, American Chemical Society. c) Schematic illustration of CO_2_ electroreduction coupled with the methanol electrooxidation reaction to formate in a flow cell, and d) corresponding (FE) towards formate measured at a current density of 200 mA cm^−2^ over 20 h, and of 1 A cm^−2^ over 3600 s. Reprinted with permission from Ref. [31] Copyright 2024, American Chemical Society.

Electrolytic cells for the CO_2_RR coupled with the electrooxidation of alcohols or sugars reported in existing literature are usually H‐type cells and flow cells. The typical H‐type electrolytic cell consists of a cathode chamber and an anode chamber. In a report by Liu et al.^[^ ^105^ ^]^ the CO_2_RR and the methanol oxidation reaction (MOR) were evaluated as cathode and anode reactions, respectively, in a H‐type cell with BiO_2_ and CuS as electrode materials, respectively. During the electrolysis process, the results from linear sweep voltammetry indicated that the coupling of the two reactions led to a reduction in cell voltage compared to coupling the OER as anode reaction. Specifically, the cell voltage in the MOR‐integrated configuration was reduced by 272 mV at a current density of 10 mA cm^−^^2^ compared with the case where the OER was the anode process (**Figure** **5**a,b).

Due to the low solubility of CO_2_ in water‐based electrolytes and a long diffusion path (≈50 μm) in H‐type electrolytic cells, the reaction is strongly affected by mass transfer limitations, and it is difficult to reach an industry‐relevant current density (>200 mA cm^−2^). The relatively long distance between the anode and the cathode leads to large ohmic losses, thereby resulting in a large energy consumption to overcome the conductivity limitations.^[^ ^131^ ^]^

Therefore, while the H‐type electrolytic cell is suitable for screening catalysts and electrolytes on a laboratory scale, researchers have designed and developed flow electrolytic cells with the aim of overcoming mass transfer limitations. A typical flow cell achieves this by means of a circulating pump to transport reactants to the cathode compartment and efficiently remove products from the reaction chamber. This design not only decreases the ohmic resistance and improves energy efficiency but also enables continuous and stable operation. For example, Liu et al.^[^ ^31^ ^]^ evaluated the performance of an electrochemical flow cell comprising the coupled CO_2_RR//MOR reactions, which achieved the industry‐relevant current density of 1.2 A cm^−2^. Importantly, the observed performance, in terms of current density and selectivity, was maintained at 200 mA cm^−2^ over a 20 h operation period (Figure 5c,d).

It is important to note that, compared with independently operated CO_2_RR or alcohol/sugars electrooxidation reactions, coupled systems often face more complex reaction environments. The cathode and anode processes may have cross effects, for example, the cathode and anode products may migrate through the ion exchange membrane to the opposite compartment, interfering with the stable operation of the counter reaction. Additionally, pH fluctuations during the cathode reduction process may change the state of the anode reaction interface, and vice versa. Thus, systematic research on electrolyte optimization and catalyst deactivation in the coupled electrolysis systems are required to further advance the electrolysis of CO_2_ coupled to the electrooxidation of alcohols and sugars. Furthermore, to achieve scalability development of specialized flow reactor designs is needed, addressing current issues such as insufficient mechanical and chemical stability not only of the electrodes but also of the membrane, gas–liquid flow management, and ion migration limitations, among others.

### 2.4 Separation and Purification of Formate

The generation of formate via coupled CO_2_RR and electrooxidation of alcohols or sugars is promising in terms of current density, overpotential, and FE. In spite of this, one important disadvantage is that the obtained product, namely formate, is diluted in the electrolyte, which makes it necessary to employ energy‐intensive separation processes to extract it from the electrolyte solution. It is estimated that the cost of separating and purifying formate from the electrolyte accounts for more than 70% of the total production cost.^[^ ^48^, ^132^ ^]^ For that reason, different approaches have been explored to reduce downstream separation energy consumption.^[^ ^133^ ^]^ Relevant approaches are summarized in this section.

A promising approach to directly obtain formic acid in the laboratory is to insert an additional solid electrolyte compartment between the anode and cathode. In this electrolyzer design, the cathode and anode are in direct contact with an AEM and a CEM, respectively. The ions produced at the cathode and anode (HCOO^−^ and H^+^) migrate through the AEM and CEM to the central compartment, where pure formic acid molecules are formed and recovered without further separation and purification.^[^ ^134^, ^135^, ^136^ ^]^ Another example is found in a work by Fan et al.^[^ ^137^ ^]^ who employed an all‐solid‐state electrochemical CO_2_RR system (with an electrode geometric area of 1 cm^2^) for continuous generation of high‐purity and high‐concentration formic acid solutions. The generated formic acid can be effectively removed in the form of a vapor by flowing an inert gas through the solid electrolyte layer. In addition to this, experimental results show that a highly active (current density ≈450 mA cm^−2^), highly selective (maximum FE ≈97% towards formate), and highly stable (100 h) CO_2_ electroreduction system can be obtained using a grain boundary enriched Bi catalyst. An ultrahigh concentration of formic acid solution (up to nearly 100 wt.%) can be condensed from the generated vapor by adjusting the carrier gas flow. Similarly, Zeng et al.^[^ ^138^ ^]^ prepared a 3 cm^2^ proton‐conducting solid electrolyte to avoid mixing of formate and electrolyte solution. The formate ions generated at the cathode (from CO_2_ electroreduction) reached the intermediate chamber under the action of the electric field, while the hydrogen protons generated at the anode migrated to the intermediate chamber to form formic acid and diffuse through water. When the deionized water flowrate is 45 mL h^−1^, the selectivity towards formic acid can be maintained above 90%. When the applied voltage is −3.86 V, the FE towards formic acid reaches 94% at a current density of 375 mA cm^−2^, resulting in a 0.16 M formic acid solution. In addition to this, the coupled electrolysis system can operate stably for 180 h at an applied cell voltage of −3.45 V.

Although the use of solid‐state electrolytes has been reported as a method to separate the product (formic acid or formate), the application of this technology on an industrial scale still faces several challenges, such as the need to further increase the electrode area and to improve long‐term stability. For that reason, other separation processes are often required to extract the product from the electrolyte. A commonly used method for separating formic acid and water mixtures industrially is distillation. Regular atmospheric distillation is not feasible to separate formic acid from an aqueous solution because of the small differences in their boiling points (100.8 and 100 °C, respectively). Since water and formic acid form an azeotrope, extractive azeotropic distillation, pressure swing distillation and salt‐addition distillation technology can be selected. Among them, the pressure swing distillation process is widely used since it is particularly suitable for the separation of azeotropic systems and does not require the addition of a third component in the mixture. Additionally, it can be combined with and electrolysis with heat integration. The pressure swing distillation process utilizes the characteristics that the composition and boiling point of formic acid and water will change under different pressure conditions, so as to perform distillation under high pressure and low‐pressure conditions to separate formic acid and water. Nonetheless, these approaches are energy‐intensive and thus costly, with a separation energy consumption of formic acid on a commercial scale of about 35 MJ kg^−1^ formic acid.^[^ ^139^, ^140^ ^]^

Electrodialysis can be used in water desalination, acid recovery, and wastewater treatment and is currently researched as an efficient alternative to extract formic acid from the electrolyte solution.^[^ ^141^, ^142^, ^143^, ^144^, ^145^ ^]^ For example, Wu et al.^[^ ^146^ ^]^ proposed to conduct electrodialysis using two different anion‐exchange membranes (3362 W and AM‐203). The electrodialyzer has an anode chamber and a cathode chamber, which are separated by an AEM, and the initial formic acid concentration in both chambers is 30 wt%. Formate anions enter the anode compartment through the AEM and combine with protons at the anode compartment to form formic acid; in the cathode compartment, H^+^ is retained and reacts with hydroxide ions to form water. The formic acid concentration in the anode compartment gradually increases, while the formic acid in the cathode compartment gradually decreases. At the same time, the cathode electrolyte can be further recycled. In another report, Kaur et al.^[^ ^144^ ^]^ presented a scalable three‐compartment electrolyzer for the CO_2_ electroreduction to formate and the extraction of formate as pure formic acid by electrodialysis. The results showed that the formic acid recovery rate was 88% using conventional electrodialysis (CED) and 46% using bipolar membrane electrodialysis (BMED). In addition, BMED could recover >95% of K^+^ as base and 12 L of pure CO_2_. The low formic acid recovery using BEMD may be due to the high alkalinity and complex chemical composition of the electrolyte solution (KOH, KHCO_3_ and K_2_CO_3_). In addition to this, CO_2_ is generated because CO_3_^2−^ or HCO_3_^−^ ions are formed during the electrochemical conversion process in alkaline media, which will be converted into pure CO_2_ when combined with H^+^ during the product separation process of BEMD, resulting in product leakage, thereby reducing recovery and increasing the extraction process and power consumption. Considering the low formic acid recovery rate and the high KOH recovery rate of the BEMD method, it is suggested to couple BMED with CED so that the overall electrodialysis process has certain industrial potential, but further research is needed to optimize the electrodialysis system and its operation conditions to achieve large‐scale formic acid production and extraction performance, as well as to improve the recovery rate.

In the case of the coupled electrolysis system comprising the CO_2_ electroreduction and the alcohol/sugar electrooxidation, the goal is to achieve a high current density and high FE towards formate at a low cell voltage, as well as achieve long‐term stability. Highly efficient anode and cathode catalysts as well as suitable electrolytes, ion exchange membranes and separation process should be further developed to optimize the electrolysis process as a whole.

## 3 Performance Assessment

In this section, the economic feasibility and environmental impact of the CO_2_RR coupled with alcohol/sugar electrooxidation are systematically evaluated, which is essential to assess the paired electrolysis process in terms of sustainability. First, we summarize and compare the effects of key parameters, such as current density, voltage, and electricity price, on the efficiency of the electrolysis process, production cost, and energy consumption, and quantitatively evaluate the economic feasibility of different anode reactions. Specifically, the production cost of formic acid via the CO_2_RR is evaluated, and the effects of different factors, such as current density, electrolyte type, and electrode material, on the production cost of formic acid are discussed. We further explore the carbon emission reduction potential under different energy supply conditions (such as fossil energy vs. renewable energy) to comprehensively evaluate the environmental advantages of the coupled system as a performance indicator to quantify the degree of improvement in its overall environmental impact. Finally, we summarize the impact of global warming impact (GWI) when the alcohol/sugar electrooxidation replaces the OER as the anode reaction.

### 3.1 Economic Performance Assessment

**Figure 6:**
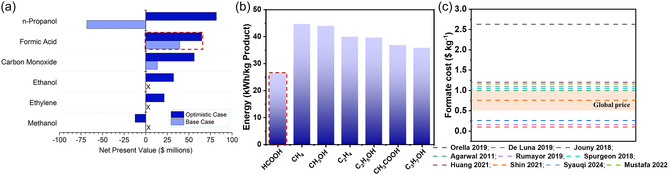
a) Net present values (NPVs) for the production of various chemicals; Reprinted with permission from Ref. [152] Copyright 2018, American Chemical Society. b) Energetic assessment of CO_2_ electroreduction towards different products; used with permission of the Royal Society of Chemistry from Ref. [153] permission conveyed through Copyright Clearance Center, Inc. c) Overview of the costs for the CO_2_ electroreduction towards formate from technoeconomic analyses in the literature;^[^ 152, 154, 155, 156, 157, 158, 159, 160, 161, 162 ^]^ the shaded area in the picture represents the global price.

Technoeconomic analysis (TEA) is a method for evaluating the technical performance and economic feasibility of a technology solution. To date, many studies have applied TEA to CO, formate, and ethene as products from the electroreduction of CO_2_.^[^ ^147^, ^148^, ^149^, ^150^, ^151^ ^]^ For example, Jiao et al.^[^ ^152^ ^]^ calculated the net present value (NPV) of various CO_2_ electroreduction products for the production of 100 t day^−1^. The NPV represents the difference between the present value of cash inflows and the present value of cash outflows over a specific period of time. An NPV greater than 0 indicates that a project is profitable. The results show that under current technical and economic conditions, formic acid is an economically viable product, with a NPV of $39.4 million (**Figure** **6**a), which positions this compound as one of the main CO_2_RR target products. Kibria et al.^[^ ^153^ ^]^ used an estimation of energy requirements to evaluate the process of CO_2_ electroreduction towards the production of eight different compounds. It can be seen from the report that the energy per kilogram required to produce different products varies, with formate (26.36 kWh kg^−1^) requiring less energy than methane (44.63 kWh kg^−1^) and methanol (44.96 kWh kg^−1^) (Figure 6b). In addition to evaluating the CO_2_RR using energy requirements, other literature reports evaluate the cost of producing 1 kg of formate. Figure 6c showed the costs of CO_2_ electroreduction towards formate production as calculated from TEAs in the literature.^[^ ^152^, ^154^, ^155^, ^156^, ^157^, ^158^, ^159^, ^160^, ^161^, ^162^ ^]^ The current price of formate reported in existing literature was 0.5–1.0 $ kg^−1^.^[^ ^41^, ^161^ ^]^ As can be seen from Figure 6c, formate is a cost‐competitive product. The production costs of formate according to the literature are close to the global price, and, under optimized electrolysis conditions, it could be even lower than the global price.^[^ ^152^, ^154^, ^155^, ^156^, ^157^, ^158^, ^159^, ^160^, ^161^, ^162^ ^]^

**Figure 7:**
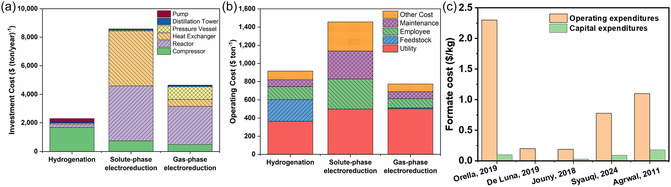
a,b) Detailed breakdown of capital and operating costs for CO_2_ reduction to formate in three different methods; Reprinted from Ref. [161] Copyright 2024, with permission from Elsevier. c) Cost breakdown for the production cost of formate with the data from the five TEAs analyzed.^[^ 152, 154, 155, 156, 161 ^]^

It is important to note that the production costs of formate calculated by different literature reports vary greatly, which requires further analysis and evaluation of the results of the technical and economic assessment process. In general, the average electrolysis cost of formate production is about 1.5 times the average market price of formate. The total formate production cost is usually calculated based on the capital cost (CapEx) and operating cost (OpEx). The CapEx contains the total direct costs, total indirect costs, and fixed capital costs, while the OpEx includes utility costs, salaries, maintenance, laboratory expenses, insurances, and feed costs (**Figure** **7**a,b).^[^ ^161^ ^]^ By analyzing the different investment and operating costs of formate according to the aforementioned literature reports, it was found that the operating costs were generally higher than the capital costs. This is mainly due to the electricity consumption required for conversion and the costs of formate separation.^[^ ^152^ ^]^ De Lunna et al.^[^ ^155^ ^]^ found that electricity prices and separation processes accounted for 65.8% and 15.9% of the total production cost, respectively. Meanwhile, Orella et al.^[^ ^154^ ^]^ reported that the separation cost of formate accounts for about 70% of the total formate production cost, while the electricity consumption accounts for about 8% of the total formate production cost. The reasons for these large differences may be attributed to the use of different calculation models as well as differences in other parameters, for instance, electricity prices. Nevertheless, it can be summarized that the electricity consumption and formic acid separation costs are the main cost drivers of the operating costs (Figure 7c).

**Figure 8:**
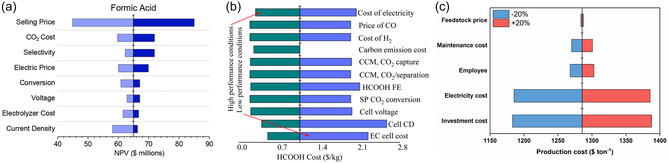
a) Sensitivity analysis of end‐of‐life NPV for CO_2_ electroreduction to formate. Reprinted with permission from Ref. [152] Copyright 2018, American Chemical Society. b) Sensitivity assessment of CO_2_ electroreduction to formate (CCM: capital cost multiplier; FE: Faradaic efficiency; SP: single‐pass; CD: current density; EC: electrochemical). Reprinted with permission from Ref. [162] under a CC BY‐NC 4.0 License. c) Sensitivity analysis of different parameters of CO_2_ electroreduction on the formate production cost. Reprinted from Ref. [161] Copyright 2024, with permission from Elsevier.

It is worth noting that, under industrial conditions, operating parameters, such as current density, selectivity and voltage, are constantly changing, so it is necessary to further analyze the impact of different technical indicators on formate costs and find out what are the varying operating parameters that have the greatest impact on formate production costs. By identifying these key parameters, cost control and optimization of the production process can be carried out more accurately, thereby improving the whole cost‐efficiency of the electrolysis process. In the case of the CO_2_ electroreduction, the parameters that influence the production cost of formate include the reaction rate (current density, CD), single‐pass conversion (SP), selectivity (FE), applied voltage, catalyst cost and lifetime, electricity price, electrolyzer cost and lifetime, and carbon tax (**Figure** **8**a,b). It is observed that reducing the current density leads to an increase in the cost of formate production, mainly because low current density requires electrolyzers with larger electrolysis area, thus increasing capital and operating costs. For example, based on a current density of 140 mA cm^−2^, a 50% increase in current density leads to a 0.28 $ kg^−1^ reduction in formate production costs, while a 50% decrease in current density leads to a significant 0.84 $ kg^−1^ increase in formate production costs.^[^ ^158^ ^]^ In addition to this, a higher selectivity of formate (>95%) produced via CO_2_ electroreduction reduces the total current density needed because the HER is inhibited. However, if the FE is decreased from 94 to 75%, the production cost of formate will increase from 1.16 $ kg^−1^ to about 1.4 $ kg^−1^. Reducing the electrolyzer voltage could lower the electricity consumption. For instance, a 0.5 V decrease in voltage (from 3.5 to 3.0 V) reduces the total electricity consumption by 13%. Additionally, a higher conversion results in a lower separation cost due to decreased amount of unconverted CO_2_ in the separation loop, and this, consequentially, decreased the size of the separation device.^[^ ^158^ ^]^ The fluctuations in electricity prices also have a significant impact on the production costs, because the electrolysis process and other non‐electrolysis process (CO_2_ adsorption, product separation, etc.) consume a large amount of electricity, and an increase in electricity prices will directly increase the production costs of formate. When the electricity price decreases from 0.06 to 0.03 $ kWh^−1^, the production cost of formic acid decreases from 1.16 $ kg^−1^ to about 1.04 $ kg^−1^. At the same time, the CapEx of the electrolyzers is also a key factor in the overall process. Efficient and durable electrolyzers (including electrode materials and membranes) can reduce replacement frequency and maintenance costs, thereby saving costs in long‐term operations. A direct reduction in the cost per unit of electrode area has a large effect in the total costs, highlighting the importance of employing low‐cost electrode materials. For instance, a 50% reduction in this cost can reduce the production cost of formate from 1.16 to 0.6 $ kg^−1^.^[^ ^158^ ^]^ Jouny and Mustafa et al.^[^ ^152^, ^162^ ^]^ also studied the impact of technical indicators such as current density, single‐pass conversion rate, and FE towards formate on the production cost of formate and found that these technical indicators have similar impacts (Figure 8c). In conclusion, taking all these operating parameters into consideration, actual production strategies at both pilot and industrial scales can be optimized to maximize cost‐effectiveness.

Electricity consumption is an important factor that dominates the prices of formate production from electrolysis processes. The total energy required for electrolysis process is determined by the sum of the cathodic and anodic energies (i.e., overpotentials) required for each half‐reaction. In this context, the implementation of anode reactions with lower energy requirements than the OER are expected to have a strong impact in the overall energy requirements of the electrolysis process as a whole.^[^ ^163^, ^164^ ^]^ Therefore, TEA tools have been used to explore the coupling of CO_2_ electroreduction to alternative anode reactions.^[^ ^165^, ^166^, ^167^ ^]^ Furthermore, combining CO_2_ electroreduction specifically with the electrooxidation of alcohols and sugars can bring considerable economic benefits. For example, Verma et al.^[^ ^24^ ^]^ found CO_2_ electroreduction and glycerol electrooxidation to be a promising coupled system, reducing electricity consumption by 53% compared to using the OER as anode reaction. At the same time, formate was obtained as a product of the glycerol electrooxidation, further improving the economic efficiency of the overall process. Similarly, Junqueira et al.^[^ ^168^ ^]^ showed that the combination of CO_2_RR with the glycerol electrooxidation increases the energy efficiency. Compared with the CO_2_RR//OER system, the energy efficiency of the CO_2_RR//GOR is 41% higher. Moreover, Xie et al.^[^ ^166^ ^]^ reported a coupled electrolysis system involving CO_2_ electroreduction and glucose electrooxidation. In this study, it was found that, compared with the state‐of‐the‐art literature reports using the OER as the anode reaction, the energy density of the CO_2_RR and glucose electrooxidation coupled system is reduced from 485 to 262 GJ ton^−1^, a decrease of about 46%. Electrons are used in both cathode and anode reactions, allowing the formation of two value‐added products with the same energy consumption, thereby increasing the product value per kilowatt‐hour by almost 4 times.^[^ ^167^ ^]^ According to the above preliminary assessment, it can be seen that the CO_2_ electroreduction coupled with an alcohol/sugar electrooxidation process is still economically attractive in terms of cost reduction based on existing experimental data, and this strategy is compatible with today's most efficient electrolyzers and catalysts. Na et al.^[^ ^37^ ^]^ used a fully automated process synthesis framework to calculate the economics of 16 cathodic reactions (the CO_2_ electroreduction towards different products, and the HER) and 18 anodic reactions (the electrooxidation of organic compounds, and the OER) coupled with each other. The results show that the profitability index depends on the type of electrooxidation reaction and not on the type of CO_2_ electroreduction.

The existing reports emphasize that the electrooxidation of alcohols and sugars, replacing the OER, can reduce the potential of the electrochemical reaction process and improve energy efficiency. At the same time, some literature has reported alcohol/sugar electrooxidation coupled with the HER to explore the effects of different operating parameters on the anodic process.^[^ ^169^, ^170^, ^171^, ^172^ ^]^ For example, Mitsos et al.^[^ ^173^ ^]^ showed that, because the thermodynamic potential of glycerol electrooxidation is lower than that of OER, conducting the GOR instead of the OER can reduce by 50% the energy consumption of the electrolysis process to produce hydrogen. Moreover, from the GOR, other commercially expensive products (such as glyceric acid and dihydroxyacetone) can be obtained, which makes it more economically favorable than generating O_2_. In 2024, Khan et al.^[^ ^172^ ^]^ calculated the economics of the process of producing formic acid from the glycerol electrooxidation coupled with the HER in an alkaline environment. The results showed that in alkaline electrolytes, further sulfuric acid acidification steps are required to obtain pure formic acid products during the GOR. If the K_2_SO_4_ obtained in the acidification process is sold as a by‐product, the production cost of formic acid is 1.57 $ kg^−1^, which is significantly higher than the market price of 1.0 $ kg^−1^. The high cost is mainly attributed to the separation cost of formic acid, which accounts for about 66% of the total capital cost. In addition to this, the electricity consumption of the formic acid product separation also accounts for 68% of the total electricity consumption. In order to reduce the production cost of formic acid, sensitivity analysis was used to explore the impact of different operating parameters on the product cost of glycerol electrooxidation to formate. The results show that reducing voltage, increasing selectivity, increasing current density, reducing the cost of KOH, and increasing the cost of K_2_SO_4_ can effectively reduce the production cost of formic acid. For example, when the selectivity of formic acid increases from 50 to 75%, the output of K_2_SO_4_ and formic acid products will increase accordingly, ultimately reducing the production cost of formic acid from 1.57 to 1.2 $ kg^−1^. Furthermore, the production cost of formic acid from the GOR is similar to that of CO_2_RR to formic acid. Both are affected by the interaction of different parameters such as operating conditions, electricity consumption, raw material prices, and by‐product value. In short, by pairing two electrocatalytic reactions, value‐added formate product can be produced at both sides and the overall system requires lower potentials, which can significantly reduce energy consumption and eventually become an economically viable system, provided that current density, high conversion and high selectivity are achieved.

### 3.2 Environmental Performance Assessment

**Figure 9:**
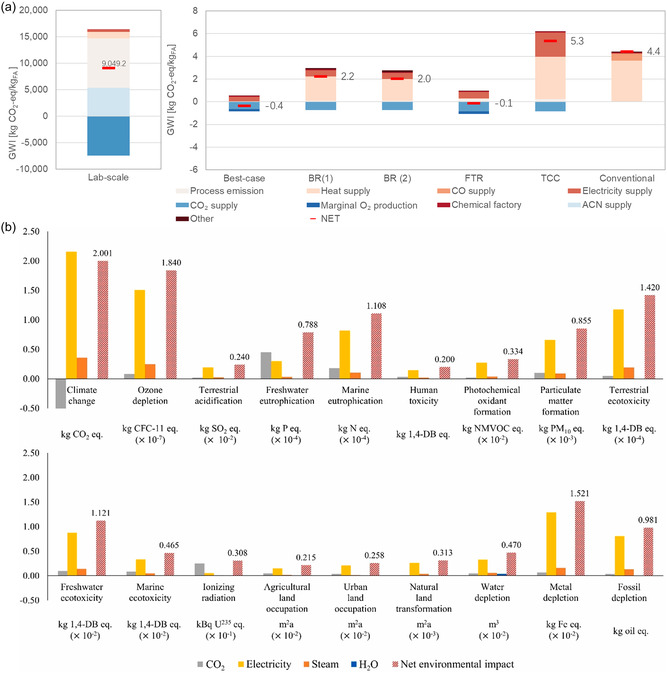
a) Analysis of the global warming impact (GWI) of the lab‐scale, upscaled, and conventional process for the production of 1 kg formate (85 wt%); BR(1): batch reactor using conventional rectification; BR(2): batch reactor using extractive rectification with benzyl formate; FTR: flow‐through reactor; TCC: three compartment cell. Reprinted with permission from Ref. [177] Copyright 2019, American Chemical Society. b) Environmental impact of CO_2_ electroreduction to formate. Reprinted from Ref. [178] Copyright 2021, with permission from Elsevier.

The CO_2_RR contributes to curbing the rise of CO_2_ concentration in the atmosphere, either by utilizing CO_2_ already present in the atmosphere or by preventing further emissions of CO_2_ from industrial processes. It is therefore important to consider the environmental impact of the coupled process in addition to the economical assessment. Life cycle assessment (LCA) is a systematic approach that allows assessing the environmental impact of formate product throughout its entire life cycle, from raw material sourcing and production, to usage and final disposal. Therefore, LCA studies of CO_2_ electroreduction processes often include environmental impact categories related to global warming.^[^ ^174^, ^175^, ^176^ ^]^ Although the greenhouse gas emissions from the CO_2_ electroreduction to formate products reported in the existing literature range widely, from 32 to 519 kg CO_2_ per kg formate, the values are generally much higher than those found for commercial formate production process (3.1 kg CO_2_ per kg formate).^[^ ^140^ ^]^ One important factor influencing this is the origin of the electricity used for the electrolysis process, which differs greatly if it is based on fossil fuels or on renewable energy sources. For example, De Luna et al.^[^ ^155^ ^]^ reported that the GWI of the CO_2_RR to produce formate powered with renewable energy was only 2.0 CO_2_ per kg formate at a power plant capacity of 500 MW. Meanwhile, Thonemann et al.^[^ ^177^ ^]^ found that, compared with traditional formate production, the same process under optimal industrial‐scale conditions has a smaller environmental impact of about −0.4 kg CO_2_ per kg formate (**Figure** **9**a).

In addition to considering the impact on climate change, Kang et al.^[^ ^178^ ^]^ also considered the evaluation of 18 environmental indicators during the CO_2_ electroreduction to formate. The results of the evaluation showed that this process has a small impact on all environmental impact factors (such as, terrestrial acidification, freshwater eutrophication, marine eutrophication, etc.), except terrestrial ecotoxicity (Figure 9b). Electricity accounts for 83% of the total terrestrial ecotoxicity in the process of electrochemical CO_2_RR to formate. Therefore, the source of electricity may become a bottleneck for environmental impact, which will be greatly reduced by using renewable electricity sources. The environmental impact of the electricity used in the electrochemical production of formate from CO_2_ was determined by selecting different electricity sources (e.g., oil, natural gas or hydropower). Compared to natural gas, oil as the electricity source showed the highest growth rates in most impact categories (minimum: 27.8% in metal depletion, and maximum: 954.4% in terrestrial acidification), while hydropower showed the lowest growth rates (minimum: −14.1% in ionizing radiation, and maximum: −107.1% in climate change). Furthermore, Paulillo et al.^[^ ^149^ ^]^ conducted a LCA of CO_2_ electroreduction to formate in an ionic liquid electrolyte. The results showed that the recovery rate of the electrolyte (ionic liquid, acetonitrile and water) greatly affects the environmental impact of the whole process. When the recovery rate of the electrolyte is 95%, the environmental impact of the electroreduction process is 2.5 to 50 times that of the traditional process. However, when the recovery rate is increased, the environmental performance is significantly improved. When the recovery rate of the process is increased to 99%, the process becomes environmentally competitive compared with the traditional route.

There are reports on the environmental assessment of the electrooxidation of alcohols and sugars. For example, Regalado‐Méndez et al.^[^ ^179^ ^]^ used fossil energy as the power source and compared the OER with the glycerol electrooxidation. In the case of the former, the associated GWI was 29.54 kg CO_2_ per kg H_2_, while integrating the glycerol electrooxidation as the anode process reduced this value to 23.04 kg CO_2_ per kg H_2_. The GWI of the GOR using fossil fuels and solar photovoltaic as power sources was also compared. If solar energy was used as the power source, the GWI was only 1.81 kg CO_2_ per kg H_2_, a decrease of 92.1%. It was also found that the glycerol electrooxidation process affected MAE (marine aquatic ecotoxicity) and ADP (fossil fuel), mainly due to the consumption of fossil energy as electricity. Therefore, it can be concluded that in order to reduce the impact on the environment, the use of non‐renewable energy should be avoided as much as possible. The environmental impact of electrooxidation of alcohols and sugars and the CO_2_RR should be considered in future studies, which may also help improve further the environmental impact.

## 4 Summary and Outlook

Feasible CO_2_ electroreduction to value‐added products, including formate/formic acid, is highly needed to address the global warming problem, thereby searching for alternative, environmentally friendly routes to utilize this compound. At the same time, in order to improve the energy efficiency of this electrolysis process, coupling the CO_2_ electroreduction with an electrooxidation reaction alternative to the conventional OER, particularly the electrooxidation of alcohols and sugars, which can be derived from biomass, enables the production of formate in both cathode and anode with reduced energy input requirements and costs. In this review, we provided a systematic overview of the recent progress of CO_2_ electroreduction coupled with the electrooxidation of alcohols and sugars towards the production of formate and evaluated the existing literature on technical, economic, and environmental aspects of the (coupled) processes. Although the conventional CO_2_ electroreduction system (with the OER as anode process) has been widely studied for many years, the coupled electrolysis system is a novel emerging research area, and many challenges remain to be addressed. Some of the challenges that should be solved in the future are summarized as follows.

### 4.1 Design of Efficient Electrocatalysts.

Catalysts play an important role in the performance of electrochemical systems, directly affecting reaction efficiency, stability, and product selectivity. The density and stability of active sites can be increased by designing nanostructured or alloy, multimetallic catalysts. In particular, Bi‐ and Sn‐based cathodes as well as Ni‐ and Co‐based anodes can be further optimized to enhance the performance of the two half reactions (CO_2_RR and electrooxidation of alcohols/sugars, respectively), targeting a formate selectivity above 90%, current density higher than 200 mA cm^−2^, and CO_2_ single‐pass conversion higher than 50%. We recommend the combination of in‐situ/online analysis, particularly spectroelectrochemistry, with computational simulations, which can be useful tools to monitor and elucidate the changes that catalysts undergo during the reaction, and can further reveal the reaction mechanism, as well as the evolution of catalysts and their performance under operation. At the same time, the design of catalysts specifically suitable for coupled systems is essential to ensure that the two electrodes can operate under compatible conditions (i.e., pH, flow rate, voltage, cell geometry, etc.) with sufficient long‐term stability. In addition to this, industrial‐scale implementation also needs to consider the design of highly active, low‐cost catalysts based on abundant, accessible, and scalable materials.

### 4.2 Electrolyzer Configuration and System Integration for the Coupled Electrochemical System.

Optimizing the flow cell configuration to ensure the stability of the coupled electrolysis process is an urgent and challenging issue that needs to be addressed in future work. The use of membranes should be comprehensively considered based on specific formate production requirements. We identify areas of opportunity, including exploring the synergistic effects of different pH environments on CO_2_ reduction and alcohol/sugar oxidation, and studying the role of bipolar membranes or selective ion exchange membranes in reducing byproduct migration between anode and cathode. Additionally, while the stability of existing coupled systems has been reported for periods of up to 100 h, further optimization is required to achieve a reaction stability for longer periods, which in turn requires in‐depth understanding of deactivation mechanisms such as poisoning, corrosion, particle agglomeration, and changes in morphology. Furthermore, it is important to consider that the separation of formate products has a substantial impact on the economic feasibility of the electrolysis process, and thus further design and development of efficient, low‐energy separation methods is urgently needed. At the same time, the interaction between coupled electrolysis technology and renewable energy supply has to be explored in depth. Considering the increasing proportion of renewable energy such as solar and wind energy in the overall energy supply, if the cost of electricity from renewable energy is less than 0.04 $ kW h^−1^ and the energy conversion efficiency reaches 70%, the production cost of formic acid can be less than 0.1 $ kg^−1^. Lastly, the coupled electrolysis system may need to have higher operational flexibility to adapt to the dynamic changes in electricity prices and effectively manage costs that change over time.

### 4.3 Optimize Economic and Environmental Aspects of the Coupled System.

Due to insufficient catalytic efficiency, insufficient energy conversion efficiency, low CO_2_ utilization rate, and high operational costs, the current technical cost of the coupled electrolysis system is still substantially high. The electroreduction of CO_2_ to formate cannot achieve environmental benefits beyond conventional processes under the existing energy structure and has not yet achieved large‐scale industrialization. Researchers should employ TEA methods to optimize the reaction process and product separation process, thereby allowing to identify potential routes to reduce costs. It is also recommended to conduct detailed cost‐benefit analyses of the coupled system to optimize the process flow and further decrease production and separation costs. Such analyses should include comparisons to current industrial standards as benchmarks for a more adequate assessment of the competitiveness of these emerging processes and technologies. Finally, developing more environmentally friendly and recyclable chemicals and materials to reduce the impact on the environment, conduct a comprehensive life cycle assessment to evaluate the environmental impact of the coupled process, and optimize the entire coupled electrolysis system is also recommended.

Overall, the electrolysis system comprising the CO_2_ electroreduction and the electrooxidation of bio‐based alcohols and sugars towards the production of formate has great potential for industrial application. However, to achieve large‐scale implementation of these systems, it is still necessary to overcome the aforementioned challenges in catalyst development and electrolytic cell design as well as to develop understanding of reaction mechanisms. Further, environmental friendliness and system integration should also be considered accompanied by cost‐benefit and TEA. With the continuous advancement of technology and the reduction of costs, integrated electrocatalytic system are expected to be commercialized in the future and contribute to the sustainable green development of energy.

## Conflict of Interest

The authors declare no conflict of interest.
